# The impact of the digital economy on occupational health: A quasi-experiment based on “Broadband China” pilot

**DOI:** 10.3389/fpubh.2022.1007528

**Published:** 2023-01-25

**Authors:** Fanfan Wang, Zheng Wang

**Affiliations:** School of Public Administration, South China University of Technology, Guangzhou, China

**Keywords:** digital economy, occupational health, improve, “Broadband China”, policy pilot

## Abstract

The recent years' booming digital economy can not only benefit businesses, but also be an important way to improve people's wellbeing. This paper aimed to identify the relationship between the digital economy based on the “Broadband China” pilot policy and occupational health by applying DID method. The empirical results highlight that occupational health in the pilot cities of “Broadband China” are significantly improved compared with those in non-pilot cities, indicating that the digital economy can significantly improve occupational health. However, this effect varies across regions with different levels of economic development. Compared with developed areas, the digital economy has a more obvious effect on the improvement of occupational health in less developed areas, which indicates that this effect conforms to the law of diminishing marginal effect. Therefore, the digital economy should be continuously promoted to improve occupational health. Governments at all levels should strengthen the network infrastructure to provide a good basic environment for the development of the digital economy. At the same time, governments should introduce more detailed digital economy goals and programs according to the actual situation of their jurisdictions to fully release the economic and social benefits of the digital economy.

## Introduction

This paper aims to investigate whether the digital economy (measured by the “Broadband China” pilot policy since 2014) has an impact on occupational health (measured in reverse as the death rate per 100 million yuan of GDP from occupational safety accidents and the number of deaths from occupational safety accidents). The concept of the digital economy was formally proposed in the Emerging Digital Economy report released by the US Department of Commerce in 1998. Different organizations and countries further interpreted the digital economy based on their own external environment and other factors. The OECD considers the digital economy to be goods and services traded using e-commerce. Major industrialized countries in the world have launched big data development policies in succession in the past few years to promote the development of the digital economy. In 2011, the US formulated the Big Data Research and Development Plan, marking the elevation of big data into a national strategy. In 2013, the Australian government issued the Public Service Big Data Strategy, aiming to use big data to promote reform of the public sector. In the same year, the Chinese government also attached importance to and encouraged the development of the big data industry.

**Figure 1:**
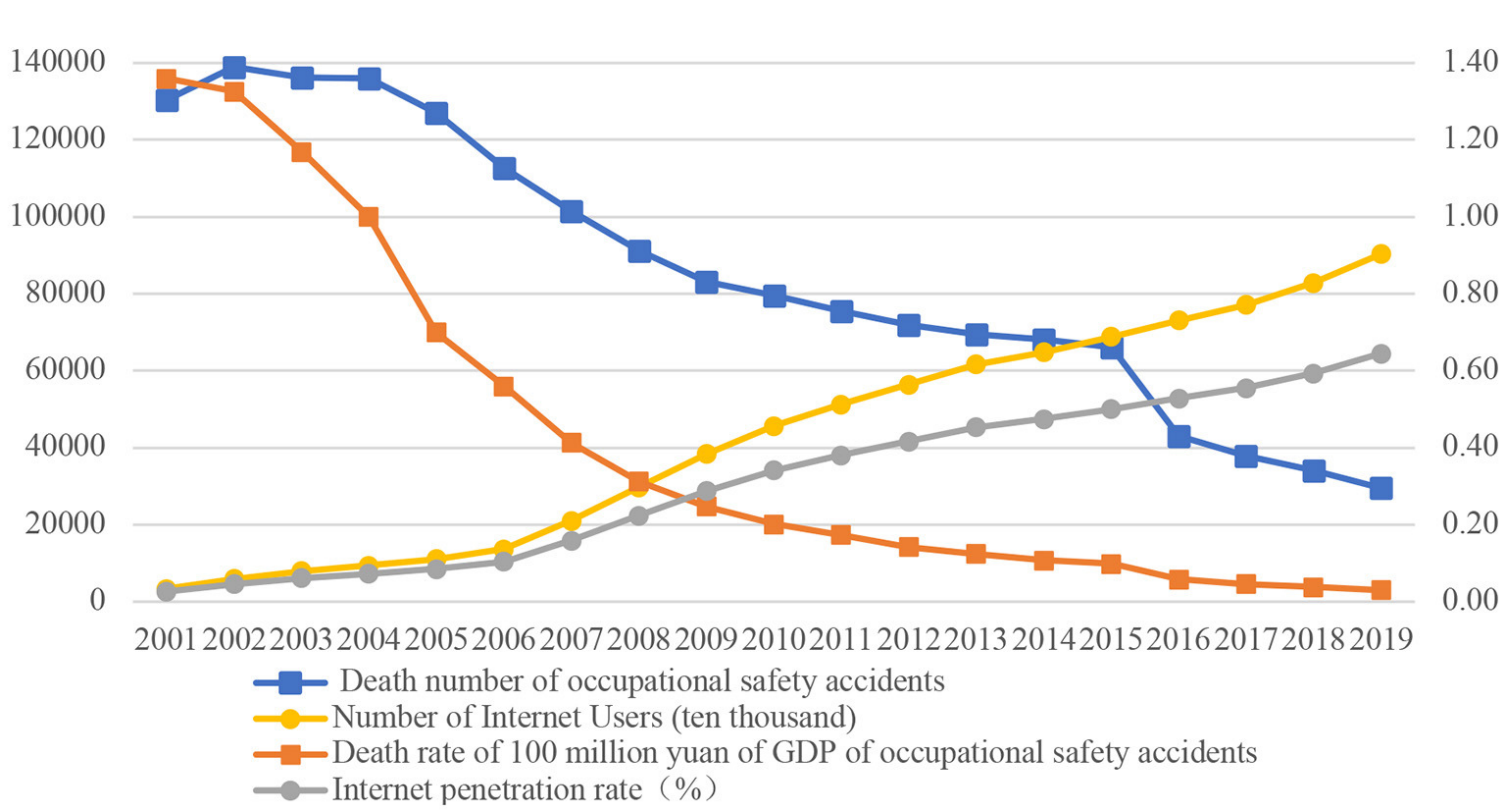
The correlationship between occupational safety accidents and internet penetration rate.

After more than 40 years of reform and opening-up, China has achieved remarkable results in its economic development. However, at the same time, accident risk in the occupational health field is also high. In 2007, there was a large gap between China and developed countries in terms of the number of deaths caused by accidents. China's death rate per 100 million yuan of GDP from occupational safety accidents is 10 times that of developed countries, and the country's accident death rate of 100,000 people in industry, mining, and commerce is more than twice that of developed countries. Figure 1 shows the relationship between the digital economy and occupational health. There is an obvious inverse relation between the internet penetration rate, which measures the development of the digital economy, and the number of deaths in occupational safety accidents. There is a similar relationship between the number of internet users and the death rate from occupational safety accidents, which implies that there may be some connection between the development of the digital economy and occupational accident. In fact, the use of the internet and the Internet of Things can better target the health needs of residents through data analysis (1). The combination of platforms such as the internet and those used by medical and health industries can improve the efficiency and reduce the costs of medical treatment. The rapid development of the Internet of Things and other fields guarantees the timely distribution of subsequent medical products, and residents can obtain systematic, full-cycle, and customized services to promote health and to improve occupational health (2).

As an important part of high-quality economic development, occupational health is related to property safety. However, economic growth and occupational health are often in conflict with each other (3). Different regions may have different preferences for economic development and occupational health, and the political, economic, and social environment in different regions may also affect the performance of digital economy benefits. Information and communication technology (ICT) has brought many economic and social dividends to humanity (4, 5), including occupational health. China's ICT industry has been developing for more than 20 years, during which time the Chinese government has introduced a series of industrial policies to stimulate investment in ICT (6). In August 2013, China released the “Broadband China” pilot program, which aims to promote the construction of broadband network infrastructure, increase the scale of broadband users, speed up broadband networks, and increase the coverage of digital networks to serve regional economic and social development. To date, China has selected 120 cities (groups) as “Broadband China” demonstration sites in three batches in 2014, 2015, and 2016, providing broad development space for China to promote digital transformation. The purpose of the “Broadband China” pilot project is to improve network transmission rate and network coverage through network infrastructure construction, so as to promote the development of national informatization and digitalization. In the 120 pilot cities, six (Guangzhou, Shenzhen, Zhongshan, Shantou, Meizhou, and Dongguan) are in Guangdong Province.

Previous studies mainly discuss the results of the digitalization of public health services (7, 8). Direct evidence that the digital economy promotes occupational health is insufficient, especially empirical studies focusing on improving occupational diseases and work-related injuries are lacking, the related intermediate mechanism also needs to be further clarified. In addition, the few empirical literatures within this research topic usually use provincial/state-level panel data and rarely use municipal-level data, thus reflecting insufficient attention to the regional heterogeneity of the digital economy in promoting occupational health.

Therefore, the question of interest in this paper is whether the rapid development of the digital economy has a statistically positive impact on occupational health. Is the “Broadband China” pilot policy effective in improving occupational health? Are the policy effects of this pilot affected by regional differences? In response to the above problems, balanced panel data from 21 cities in Guangdong Province from 2001 to 2019 were used to examine the impact of the digital economy in the “Broadband China” pilot policy on occupational health. The marginal contribution of this paper is as follows. First, the empirical results show that the “Broadband China” pilot policy significantly improved occupational health, which remained true after parallel trend tests and other robustness tests. This finding shows that the digital economy has a significant, positive impact on occupational health. Second, the influence of the digital economy on occupational health conforms to the law of diminishing marginal effects. Compared with developed areas, the digital economy has a more obvious effect on occupational health in less developed areas.

The structure of this paper is as follows. The Literature Review Section reviews the existing literature, and The Digital Economy and Occupational Health Section describes the theoretical analysis and proposes hypotheses. The Methodology and Data Section introduces the empirical method, empirical data and variables. The Empirical Results Section analyses the empirical results, and the last section is the conclusion.

## Literature review

The digital economy, as a relatively broad concept, can be included in any economic form that uses data to guide resources, to play a role, and to promote productivity. Kim et al. (9) look at it microscopically and hold that the digital economy is a new economic form, and the goods and services needed by the main body are all traded based on digital information technology. Kling and Lamb (10) argue that the digital economy is based on information for the production, supply, and distribution of goods and services from the economic sector. According to the definition given by the China Information Technology Institute, the digital economy is composed of digital industrialization and industrial digitalization, in which digital knowledge and information are the key factors in production (11). Digital technology is being widely used in modern economic activities, increasing economic efficiency and accelerating the transformation of economic structure, and becoming an important driving force for global economic recovery. For China, the digital economy is not only a new variable of economic transformation growth but also a new blue sea of economic improvement and efficiency. It is closely linked to people's lives and economic growth. Accelerating the integration of digital technology into the life services industry is important, and promoting the digitalization of the life services industry, including tourism, medical care, health care, education, film, television, and audio-visual services and games, or by combined with building construction technology in order to realize the sustainable development of low carbon development route (12, 13), these are conducive to the public health. The impact of the digital economy on occupational health has been a focus of scholars. There are many reasons for the reduction of occupational health, such as imperfect safety measures by the company, backwards health concept among the workers, and backwards property rights protection system (14). The Swiss Cheese Model reveals the relationship between unsafe behavior, unsafe conditions, and organizational management and finds the causes of accidents consists of personal factors and organizational management problems (15). Moreover, organizational management includes both internal and external management.

First, through statistical analysis of major gas explosion accident data from 1980 to 2000, to study the causes of these accidents, scholars found that 96.59% of the accidents are due to personal errors (16). A study found that workers who feel they are in danger at work do not benefit from improved safety at work because when that happens, the worker may experience greater psychological pressure, causing mistakes (17). Health promotion theory also shows that the internet can improve worker health by relieving anxiety (18). Second, the company's interior governance structure is a key factor. The lack of supervision led to frequent incidents in coal mines. However, the establishment of vertical management can reduce the probability of mine accidents (19). According to data from American companies, foreign scholars found that most work accidents occurred in companies with a high debt ratio and insufficient capital (20). Scholars also use panel data from coal enterprises and find that a high corporate debt ratio will lead to more deaths and higher occupational health risks (21). Finally, regarding the company's external factors, the government's economic policy and governance policy are an important reason, and the problems caused by the lack of property rights can explain why in many small and medium-sized coal mines in China, due to the lack of clear property rights, safety investment is insufficient, causing more than a hundred accidents (22). In addition, under the pressure of GDP assessment, the Chinese government encourages enterprises to increase their output value for financial assessment (23), leading some enterprises to choose a high-risk mode of production resulting in an reduction in occupational health (3). Governance policies also have an impact on occupational health. The government paid attention to the management of mining accidents, greatly reducing the number of casualties (24). With “one-vote veto” policies, the death rate was cut in half (20). Soft management is considered to be an important management tool for improving occupational health compared to mandatory regulations (25).

The digital economy and technology have a potential impact on improving occupational health. Previous studies have shown that the digitalization of health governance approaches can improve the efficiency of public health services (8). The digital economy and technology can also facilitate a shift in corporate management approaches, such as the provision of professional digital occupational health counseling, training and medical services (7), or directly affect individual workers by improving their personal health awareness (26) to reduce occupational health risks (27) and improve occupational health levels (28). In addition, the development of the digital industries has been shown to improve the efficiency of regional public health services (29). Although a series of studies have explored the potential relationship between digital economy and occupational health improvement, research on this topic is still insufficient, direct evidence of the digital economy and technology promoting occupational health is lacking in particularly. Meanwhile, there are few studies using urban panel data to measure the regional heterogeneity of the above effects.

## The digital economy and occupational health

The impact of the digital economy and technology on occupational health follows two main pathways. Firstly, On the hand of direct influence, digital economy and technology improves individual occupational health awareness and health level. On the other hand, digital economy and technology stimulate enterprises to adopt more scientific and healthy management methods and promote employees' health benefits, to make it possible to indirectly improve employees'occupational health.

For direct influence, Digital interventions have been shown to promote workers' occupational health perceptions and psychological attitudes by enhancing the accessibility of health information and achieving educational effects (30). A crucial factor affecting occupational health is the employees' level of technical knowledge. Employees with insufficient technical knowledge are prone to various errors, which in turn reducing occupational health. However, technical knowledge has the attributes of both explicit knowledge and tacit knowledge, which are easily influenced by spatial distance in the process of dissemination. A longer geographical distance will prevent the free flow of technical knowledge or make the flow extremely costly (31). Although the improvement of transportation infrastructure has broken the spatial distance of technical knowledge to a certain extent and promoted the flow of knowledge elements (32, 33), the transportation cost and time cost are still high and cannot guarantee completely free flow. The network infrastructure that has been gradually improved along with the development of the digital economy can rely on the flow of information elements to transmit knowledge, reduce time costs, and improve the efficiency of knowledge dissemination under the condition that no physical displacement of elements occurs, which has a profound impact on the technical knowledge reserve of enterprise employees (34). Specifically, high-skilled employees and low-skilled employees can break through the limitation of time and space to communicate with each other through instant communication and video conferences, which greatly accelerates the speed of knowledge dissemination and reduces the cost of knowledge acquisition. Therefore, the development of the digital economy can significantly improve the efficiency of technical knowledge spill over (30), which in turn improves technical support for low-skilled employees. This improvement is undoubtedly crucial for occupational health for employees.

For indirect influence path, the digital technology revolution has transformed production and R&D models, organizational structures, and ecosystems for many companies, prompting companies to rethink and innovate their corporate strategies and business models to gain sustainable competitive advantage (35). Among them, occupational health is a key factor affecting companies' competitiveness. Another alternative way for the digital economy and technology to improve occupational health may be realized by the mediating influence of the efficiency of public health services (29), and the increased accessibility and convenience of public health services is certainly beneficial to occupational health.

From the perspective of corporate employee health management, the digital economy is helpful to reduce the transaction cost of enterprises and promote occupational health. Transaction costs mainly include the cost of access to information, negotiation costs, transportation costs, contract costs, and others (36). In the digital economy, in the big data era, information flows more completely, the probability of opportunistic behavior is reduced, the transaction frequency is faster (37), and thus, the transaction cost is reduced. The change of the enterprise's mode of production and the increase in the enterprise's production efficiency change the enterprise's form. The enterprise's purchase, marketing, and management modes will also change, which will strengthen internal management and promote employees' occupational health (38). In addition, the digital economy is helpful for enterprises to learn advanced management modes and more humane management systems, to promote the progress of their own business management and to improve occupational health.

The digital economy can also improve occupational health by reducing worker operational risks. Traditional production methods mainly rely on engineers to manually control and coordinate various subsystems, which cannot avoid accidents caused by slow response and operation errors. AI services emerging from the growth of the digital economy can drive productivity gains by supplementing or replacing labor through the use of cheaper capital. Of course, while AI largely replaces low-skilled jobs, it also replaces some high-skilled jobs to some degree, but overall, AI creates some high-skilled jobs while reducing low-skilled jobs. This situation is certainly a key element in reducing occupational health risks for employees. Additionally, the use of the industrial internet, big data, and other emerging technologies can turn the traditional manual, empirical control model into standardized and precise control, while the improvement of data transmission and data processing capabilities means faster response time and more accurate operating procedures, ultimately achieving more efficient production and lower occupational health risks (39). Therefore, the digital economy has improved the productivity of enterprises while largely reducing the occupational health risks caused by the traditional production model. Based on the above three levels of analysis, we propose the following hypothesis:

Hypothesis 1: The digital economy significantly improves occupational health.

Acemoglu et al. (40). believe that for the development of technological R&D and the high-tech industry, the difference in the factor endowment of different countries and regions will also lead to great differences in the performance of technological R&D on economic growth. The initial factor endowment will lead to different effects of the same economic policy. Additionally, from the point of origin and development of the digital economy, beginning in 2014, the “Broadband China” pilot policy began with network infrastructure construction to improve the network transmission rate and network coverage to promote the development of national informatization and digitization, but as a result of each region having resource endowments, the initial levels of economic development and industrial policy are different. The policy effect of “Broadband China” differed greatly among regions. According to the basic economic principle of diminishing marginal effects, the impact of digital economic development on occupational health in economically developed areas is “icing on the cake” because of the superiority of network infrastructure and policy preferences in economically developed areas. The network infrastructure in less developed areas is relatively backwards, and the impact of digital economy on occupational health in less developed areas is “sending charcoal in a snowy day.”

As the largest economic province in China, Guangdong's economic development has been at the forefront of the country for many years. However, the problem of unbalanced regional and urban development still exists in Guangdong. Especially in the economic field, the relevant data from the Guangdong Statistical Yearbook in 2020 show that the land area of the Pearl River Delta region (PRD) only accounts for 30% of the province, and GDP in 2020 is as high as 8.94 trillion yuan, which accounts for more than 80% of the province. However, the land area of the non-Pearl River Delta region (non-PRD) accounts for 70% of the province, and GDP in 2020 was only more than 2 trillion yuan, which accounts for less than 20% of the province. There is an obvious imbalance in the economic development of Guangdong (41). Due to the more advanced development conditions of network infrastructure and technological innovation in cities and economically developed areas, according to the law of diminishing marginal effect, digital economy development based on the “Broadband China” pilot policy has less impact on occupational health in economically developed areas than in less developed areas. Therefore, the following hypothesis is proposed:

Hypothesis 2: The influence of the digital economy on occupational health conforms to the law of diminishing marginal effects. Compared with developed areas, digital economic development has a more obvious effect on the reduction of occupational health in less developed areas.

## Methodology and data

### Empirical strategy

Using a staged process, we developed a series of empirical models to explore whether the development of the digital economy can improve occupational health. We first performed a time-varying DID model to identify the impact of digital economy on occupational health. The digital economy depends on network transmission rate and network service quality (4). Therefore, “Broadband China” policy is an important support and core driving force for the digital economy development, which can be regarded as a quasi-experiment to measure digital economy. Since whether a city becomes a demonstration site for “Broadband China” mainly depends on the number of internet users and network access capacity in the city, it is logically not affected by occupational health, thus effectively eliminating the reverse causality problem. Therefore, the “Broadband China” pilot policy is regarded as a quasi-experiment to identify the impact of the digital economy on occupational health. We construct two dummy variables: one is the treatment and control group dummy variable du, the treatment group is the pilot cities of “Broadband China,” defined as 1; the control group is the non-pilot cities, defined as 0. The second is the policy time dummy variable dt, with the corresponding pilot establishment year as the policy occurrence point, set as 1 in the year and after, otherwise defined as 0. Since the pilot time of “Broadband China” does not occur in the same year, to test Hypothesis 1, we use a time-varying DID model by controlling two-way fixed effects and construct the following econometric model (42):


(1) $$\begin{array}{l} Y_{it}=\beta_{0}+\beta_{1}DID_{it}+{\sum \gamma_{j}X}_{it}+\mu_{i}+\sigma_{i}+\varepsilon_{it} \end{array}$$

where, *i* represents the city and *t* represents the year. The dependent variable *Y*_*it*_ represents the occupational health. *DID*_*it*_ is independent variable which refers to the interactive variable of the multiplication of the du and dt. *X*_*it*_ represents some other control variables, including the level of economic development (ln_*gdp*), industrial structure (*Industrial*), fiscal decentralization (*Fiscal*), fixed asset investment (*Invest*), foreign direct investment (*Fdi*), medical level (*Medical*), and transition effects (*Change*). μ_*i*_ represents the unobserved factors that do not change with time in each city to control the regional fixed effect. σ_*i*_ controls the time fixed effect. ε_*it*_ is the random perturbation term. For the abovementioned model, we pay more attention to the estimate of the coefficient β_1_, which measures the influence of the digital economy on occupational health. If the “Broadband China” pilot policy improves occupational health, then the coefficient of β_1_ should be significantly negative.

The underlying assumption for the DID estimator is that treatment and control cities have parallel trends in the outcome before “Broadband China” pilot policy implementation. To test this assumption, we conduct a parallel trend test following Beck et al. (43):


(2) $$\begin{array}{l} Y_{it}=\beta_{0}+\overset{M}{\underset{m=k,m\neq -1}{\sum }}DID_{it,k}^{*}\beta^{k}+{\sum \gamma_{j}X}_{it}+\mu_{i}+\sigma_{i}+\varepsilon_{it} \end{array}$$

where *Broadband*_*it, k*_ is a series of dummy variables representing the treatment status at different periods. The dummy for *m*= −1 is omitted in Equation (2) so that the post “Broadband China” pilot policy effects are relative to the period immediately before the launch of the pilot policy. The parameter of interest β^*k*^ estimates the effect of the “Broadband China” pilot policy *m* year after establishment. Because the “Broadband China” pilot policy is not in effect in all cities at the same time, *k* represents different years for different cities. Intuitively, the coefficient β^*k*^ measures the difference in the occupational health between cities under the “Broadband China” pilot policy in period *k* relative to the difference 1 year before the initiation of the “Broadband China” pilot policy. We expect the “Broadband China” pilot policy to improve occupational health, with β^*k*^ being positive when *k*≥ 0. If the parallel trend assumption holds, β^*k*^ would be close to zero when *k* ≤ −2.

### Data and variables

Guangdong Province is a very important economic province in China and a pioneer of reform and opening up. Since 1989, Guangdong's GDP has been the highest in China, and it has become the largest economic province in China. According to the Guangdong Provincial People's Government, the added value of Guangdong's digital economy in 2020 was approximately 5.2 trillion yuan, continuing to lead the country. Another key reason for selecting Guangdong Province as a sample is its unbalanced regional development. Guangdong Province includes the economically developed PRD regions such as Guangzhou, Shenzhen and Foshan, as well as the developing non-PRD regions such as Zhaoqing, Yangjiang, and Chaozhou. There are obvious differences in industrial layout and worker protection among different regions, which also provide general insights into the impact of the digital economy on occupational health at different levels of economic development. Therefore, our sample is well-representative. Twenty-one cities at the prefecture level and above in Guangdong Province from 2001 to 2019 were selected as the research samples.

The advantage of using municipal-level samples for empirical analysis compared to provincial-level samples is that municipal-level data can more realistically reflect the actual effects of local government governance, whereas provincial-level data is too macro to reflect the effects of local government governance. At the same time, the advantage of using a municipal level sample for empirical analysis compared to a county level sample is that data at the county level is difficult to obtain effectively, whereas data at the municipal level can be obtained more comprehensively. Considering the above two factors, we finally chose the municipal-level sample as the subject of the study. All data are obtained from the “Guangdong Statistical Yearbook” and the “Statistical Bulletin of National Economic and Social Development” of each city.

### Dependent variables

In 2004, the Work Safety Commission of China listed the number of deaths in workplace accidents in industrial, mining and commercial enterprises, the number of deaths in coal accidents and the death rate of one million tons of coal mines as occupational health assessment indicators. And included the death rate per 100 million yuan of GDP, the death rate per 100,000 people and the death rate per 100,000 people working in industry, mining and commercial commerce as the indicators for the record. With the continuous improvement of occupational health evaluation index system, the measurement of occupational health is also composed of absolute index and relative index. We measured the occupational health by using the death rate per 100 million yuan of GDP from occupational safety accidents (accident mortality rate, *Ln*_*rate*) and the number of deaths from occupational safety accidents (number of accident deaths, *Ln*_*death*). The lower the accident mortality rate and the number of deaths, the higher level of the occupational health.

### Independent variables

In 2014, Guangzhou, Shenzhen, and Zhongshan became the pilot cities of the “Broadband China” policy, and Shantou, Meizhou, and Dongguan became the pilot cities of the “Broadband China” policy in 2015. In this paper, we construct two dummy variables: one is the treatment and control group dummy variable du, the treatment group is the pilot cities of “Broadband China,” defined as 1; the control group is the non-pilot cities, defined as 0. The second is the policy time dummy variable dt, with the corresponding pilot establishment year as the policy occurrence point, set as 1 in the year and after, otherwise defined as 0. The independent variable is *DID*_*it*_ that refers to the interactive variable of the multiplication of the du and dt.

### Control variables

We control for seven socioeconomic variables (44, 45). The first is the level of economic development. Low input inadequacy and production technology levels result in frequent workplace accidents (46), stronger economic development, and improved occupational health management inputs and technical levels. We control the impact of economic development on occupational health by adding GDP. The second variable considered is industrial structure. Occupational workplace accidents have obvious industrial characteristics. Both the mining industry and the construction industry belong to industries with poor safety levels, and the fatality rate in the mining industry is much higher than average. This paper measures industrial structure by the ratio of gross output value of tertiary industry to GDP. The third variable is the level of fiscal decentralization. The Chinese fiscal decentralization system entrusts local governments with a certain degree of revenue autonomy, which is not conducive to investment in safety governance and occupational health management (47). In this paper, the fiscal autonomy index is used to measure the degree of fiscal decentralization, which is calculated by dividing budgetary revenue by budgetary expenditure. The fourth variable considered is fixed asset investment. Improving fixed asset investment can help enterprises upgrade equipment and pursue technical transformation to improve occupational health. This paper uses the proportion of fixed assets investment of regional GDP as a measure. The fifth variable is foreign direct investment and measured by the proportion of actually utilized foreign direct investment in GDP. The sixth variable is medical level, which is measured by the number of hospital beds per 10,000 people. The seventh variable considered is the transition effect. Under the cadre appointment system, officials at all levels in China transition in relatively fixed years (48), so the 4 years of transition of 2002, 2007, 2012, and 2017 are further controlled to investigate the impact of political cycles on occupational health. To ensure the accuracy of the measurement results, accident mortality rate, number of accident deaths and the level of economic development taken as logarithms.

Descriptive statistics of the related variables are shown in Table 1. It shows that the accident mortality rate and number of accident deaths in the pilot cities are overall smaller than in the non-pilot cities. This is a preliminary indication that the digital economy based on the “Broadband China” pilot is effective in improving occupational health.

**Table 1:** Descriptive statistics of the variables.

	**Pilot city**				**Non-pilot City**			
	**Max**	**Min**	**Mean**	**Std. dev**.	**Max**	**Min**	**Mean**	**Std. dev**.
Ln_rate	0.971	−4.510	−1.829	1.266	1.163	−4.075	−1.352	1.152
Ln_death	7.771	3.605	6.036	0.860	7.053	3.891	5.411	0.633
Ln_gdp	10.27	5.277	7.870	1.296	9.34	4.578	6.741	0.949
Industrial	2.626	0.462	1.076	0.435	1.729	0.431	0.875	0.25
Fiscal	1.035	0.206	0.712	0.239	0.955	0.135	0.475	0.196
Invest	104.237	12.652	32.661	16.682	128.862	11.332	48.153	21.224
Fdi	15.100	0.186	3.294	2.890	47.770	0.048	3.711	4.621
Medical	65.390	11.420	31.130	13.060	63.210	9.032	29.770	12.790
Change	1.000	0.000	0.211	0.409	1.000	0.000	0.211	0.408

## Empirical results

### “Broadband China” policy pilot and occupational health

Using a time-varying DID model to identify causes, the regression results of dependent variables are often affected by control variables, which adversely affects the empirical results. To enhance the robustness of the empirical results, Columns (1) and (2) and Columns (3) and (4) in Table 2 report the estimation results without and with control variables, respectively. The estimated coefficients of the explanatory variable for *DID* are significantly negative, which initially indicates that the pilot cities selected for “Broadband China” had significantly reduced accident mortality rate and number of accident deaths. That is, the development of a digital economy can improve occupational health. We continue to add control variables to obtain Columns (3) and (4). The estimated coefficient of the explanatory variable Broadband remains significantly negative, indicating that the impact of the digital economy on occupational health is stable. Compared with Columns (1) and (2), the size of *R*^2^ in Columns (3) and (4) is significantly increased, indicating that the model has stronger explanatory power. Based on the above results, we made a preliminary judgement that Hypothesis 1 was supported.

**Table 2:** Regress results of “Broadband China” policy pilot and occupational health.

	**ln_rate**	**ln_death**	**ln_rate**	**ln_death**
	**(1)**	**(2)**	**(3)**	**(4)**
DID	−0.243^***^	−0.265^***^	−0.295^***^	−0.299^***^
	(0.050)	(0.047)	(0.048)	(0.048)
Lngdp			−0.749^***^	0.150
			(0.118)	(0.119)
Industrial			−0.003	−0.003
			(0.003)	(0.003)
Fiscal			−0.687^***^	−0.673^***^
			(0.218)	(0.219)
Invest			−0.005^***^	−0.004^***^
			(0.001)	(0.001)
FDI			0.001	0.002
			(0.004)	(0.004)
Medical			−0.000	−0.001
			(0.004)	(0.004)
Change			−1.480^***^	−1.287^***^
			(0.268)	(0.269)
Region FE	√	√	√	√
Year FE	√	√	√	√
Constant	0.230^***^	6.153^***^	5.298^***^	5.901^***^
	(0.045)	(0.042)	(0.701)	(0.705)
*R* ^2^	0.968	0.852	0.976	0.870
*N*	399	399	399	399

*** Indicates significance at the 1% level. Standard error in parentheses.

### Parallel trend test

Satisfying the assumption of a parallel trend is one of the basic conditions for the establishment of a time-varying DID model. According to Formula (2), we will further test whether the treatment group and the control group meet the parallel trend assumption. With the help of the event analysis framework, this paper examines whether there is a parallel trend in the changes in occupational health in the treatment and control groups before the “Broadband China” pilot. Specifically, we set time dummy variables that are 1 in the current year and 0 in other years and cross-multiply them with grouped dummy variables of policy impact. These interaction terms are then estimated in regression Formula (2) based on the previous year of the policy shock. If the interaction term before the policy impact year is significant, it indicates that there is a significant difference in the change trend between the control group and the treatment group before the policy impact. Conversely, if the interaction terms before the policy impact year are not significant and the interaction terms in the current year or after the policy impact year are statistically significant, the parallel trend hypothesis is met.

**Figure 2:**
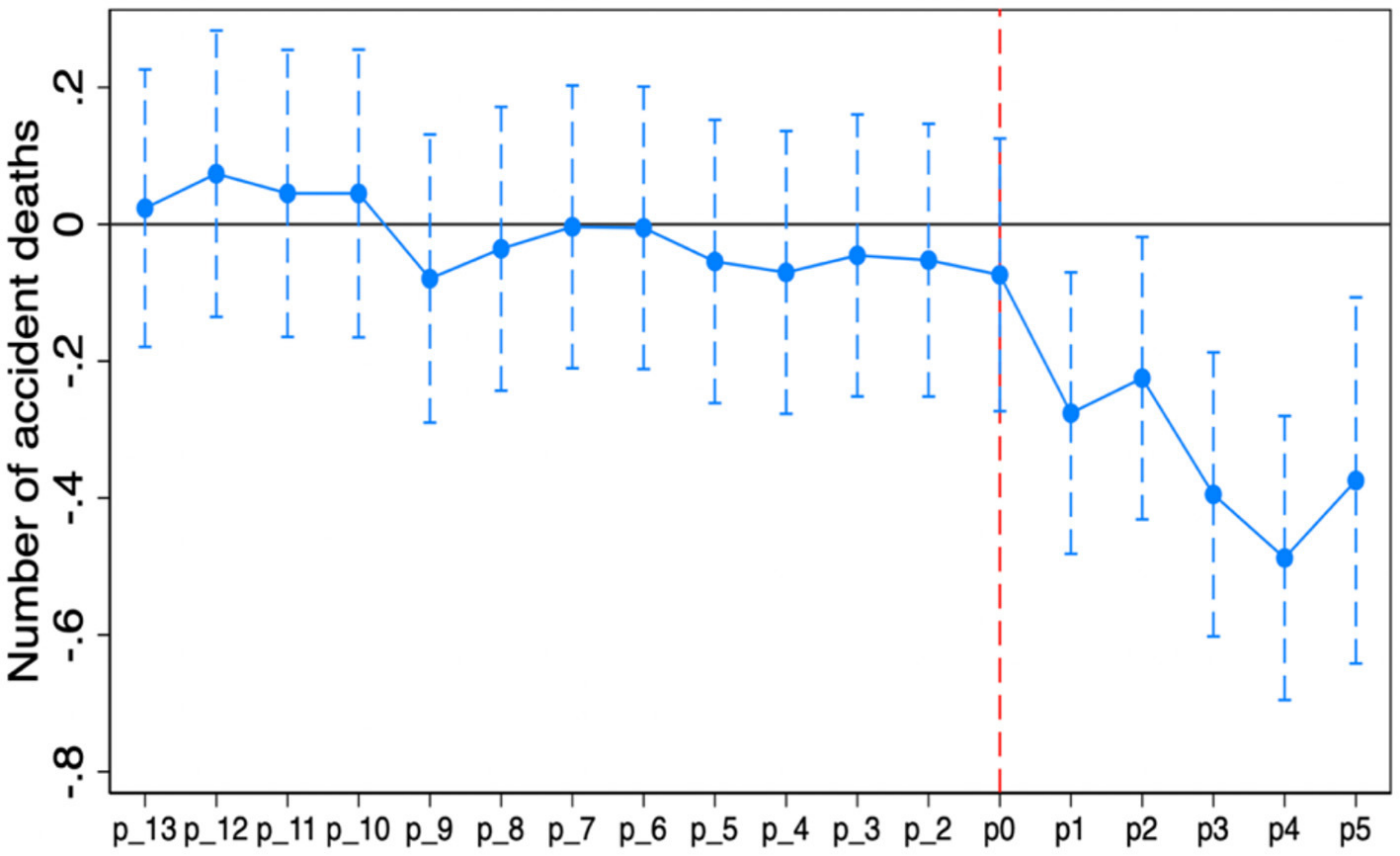
The result of dynamic effects (Number of accident deaths).

**Figure 3:**
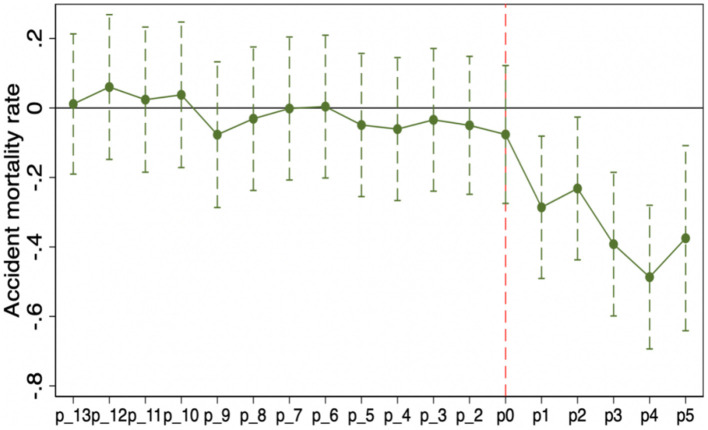
The result of dynamic effects (Accident mortality rate).

Figures 2, 3 plot the estimated coefficients and 95% confidence intervals of each interaction term with the accident mortality rate and number of accident deaths as dependent variables, respectively, and the results show that the time-varying DID model in this paper satisfies the parallel trend hypothesis and that the policy effect of the “Broadband China” pilot has obvious sustainability characteristics.

### Robustness tests

Although the estimates reported in Table 2 are relatively robust in showing that the digital economy can significantly improve occupational health, it still cannot completely eliminate the interference of issues such as omitted variables and sample selection bias. Therefore, in order to further improve the reliability of the empirical results, this paper carries out additional robustness tests.

### Replacing the independent variable

We re-measured the development of digital economy from two aspects: Internet development and digital finance development. Internet development indicators specifically use the Internet penetration rate, relevant practitioners, relevant output and mobile phone penetration rate. The corresponding actual contents are followed by the number of Internet broadband access users per 100 people, the proportion of employees engaged in computer services and software industry in urban units, the total amount of telecom business per capita, and the number of mobile phone users per 100 people. The original data of the above indicators are from China Urban Statistical Yearbook. For the development of digital finance, we use the China Digital Financial Inclusion Index jointly compiled by the Digital Finance Research Center of Peking University and Ant Financial Services Group. Through the method of principal component analysis, the data of the above five indicators are standardized and dimensionally reduced, and the comprehensive development index of the digital economy is obtained. As the China Statistical Yearbook publishes more complete indicators on Internet development from 2011 onwards, we have shortened the study interval to 2011–2019, with a total sample of 189. Table 3 reports the results of the regressions based on changing the independent variables. We find that the coefficient on the re-measured variable of the digital economy remains significantly negative, indicating that the development of the digital economy does improve occupational health.

**Table 3:** Results of replacing the independent variable.

	**ln_rate**	**ln_death**	**ln_rate**	**ln_death**
	**(1)**	**(2)**	**(3)**	**(4)**
Digital	−0.165^**^	−0.119^*^	−0.195^***^	−0.191^***^
	(0.066)	(0.066)	(0.064)	(0.065)
Control variables	×	×	√	√
Region FE	√	√	√	√
Year FE	√	√	√	√
Constant	−1.902^***^	5.443^***^	−1.321	−1.272
	(0.048)	(0.048)	(2.928)	(2.949)
*R* ^2^	0.845	0.660	0.884	0.736
*N*	189	189	189	189

^***^, ^**^ and ^*^ respectively indicates significance at the 1, 5, and 10% level. Standard error in parentheses.

### Controlling the impact of inherent city differences

To further exclude estimation bias due to non-randomness, we then control for the influence of inherent differences in cities. Because of “Broadband China,” the determination of the pilot city list is not random, and whether a city was identified as the pilot unit may be related to the city's geographic location and administrative level, which is closely related to city property and initially indicates differences between the two cities. Over time, the city's occupational health and environment may have different trends, resulting in the deviation from the estimation. To control the influence of these factors, referring to the methods of Edmonds et al. (49) and Lu et al. (50), this paper added the cross terms of these baseline factors and time linear trends into the regression and adopted the following estimation equation:


(3) $$\begin{array}{l} Y_{it}=\beta_{0}+\beta_{1}Broadband_{it}+{Z_{i}*Trend_{t}+\sum \gamma_{j}X}_{it}+\mu_{i}+\sigma_{i}+\varepsilon_{it} \end{array}$$

where *Z*_*i*_ represents the geographical location of the city and its original political and economic characteristics, including whether it is a city in the Pearl River Delta region, whether it is a coastal city, and whether it is a special economic zone. *Trend*_*t*_ represents the time-linear trend. Therefore, *Z*_i_*Trend_*t*_ indicates that the effect of the inherent characteristic differences between cities on occupational health is controlled linearly, and the estimation bias due to the non-random selection of treatment and control groups is mitigated to some extent again. We consider the impact of inherent city differences to obtain Table 4. The estimated coefficients of the core explanatory variables in all models are negative, and all are significant at the 1% level, again demonstrating that the development of the digital economy improves occupational health.

**Table 4:** Results of controlling the impact of inherent city differences.

	**ln_rate**	**ln_death**	**ln_rate**	**ln_death**
	**(1)**	**(2)**	**(3)**	**(4)**
DID	−0.236^***^	−0.293^***^	−0.274^***^	−0.283^***^
	(0.055)	(0.052)	(0.050)	(0.050)
Control variables	×	×	√	√
Region FE	√	√	√	√
Year FE	√	√	√	√
Constant	0.223^***^	6.147^***^	4.822^***^	5.484^***^
	(0.044)	(0.042)	(0.712)	(0.716)
*R* ^2^	0.970	0.858	0.977	0.878
*N*	399	399	399	399

*** Indicates significance at the 1% level. Standard error in parentheses.

### Controlling other policy effects

From 2001 to 2019, China introduced some laws and regulations at the national level to reduce local occupational safety accidents. These guiding laws and regulations are highly correlated with the dependent variables in this paper. If they are not controlled, it is difficult to ensure that the impact of the “Broadband China” pilot policy on occupational health is stable. This paper focuses on considering the influence of supervisory systems that have been implemented since 2010. In the sample period of this paper, six cities have been supervised by the central government since 2010 due to serious occupational safety accidents. The supervision system may interfere with the impact of smart city construction on occupational health. Based on Formula (1), this paper constructs the following model:


(4) $$\begin{array}{l} Y_{it}=\beta_{0}+\beta_{1}DID_{it}+\beta_{2}Supervision_{it}+{\sum \gamma_{j}X}_{it}+\mu_{i}+\sigma_{i}+\varepsilon_{it} \end{array}$$

where *Supervision*_*it*_ represents whether city *i* was supervised in year *t*, and it takes one if city *i* was supervised in year *t* and the years after and zero otherwise. The supervision system data come from the official website of China's Ministry of Emergency Management. Table 5 reports the regression results after controlling for the effects of other policies. The estimated coefficient of *DID* is still significantly negative.

**Table 5:** Results of controlling other policy effects.

	**ln_rate**	**ln_death**	**ln_rate**	**ln_death**
	**(1)**	**(2)**	**(3)**	**(4)**
DID	−0.220^***^	−0.256^***^	−0.282^***^	−0.287^***^
	(0.049)	(0.047)	(0.048)	(0.048)
Supervision	−0.151^***^	−0.059	−0.098^***^	−0.090^**^
	(0.038)	(0.037)	(0.037)	(0.037)
Control variables	×	×	√	√
Region FE	√	√	√	√
Year FE	√	√	√	√
Constant	0.230^***^	6.153^***^	4.890^***^	5.525^***^
	(0.044)	(0.042)	(0.711)	(0.716)
*R* ^2^	0.970	0.853	0.976	0.872
*N*	399	399	399	399

^***^ and ^**^ indicates significance at the 1 and 5% level. Standard error in parentheses.

### Using PSM-DID model

The select of treatment and control groups is critical in determining the accuracy of the DID estimates. This means that the control group accurately reflects the change in occupational health in the counterfactual scenario where the treatment group is in a city that is not piloting the “Broadband China” policy. To address this issue, a robustness check is conducted using a combination of Propensity Score Matching (PSM), which addresses the issue of sample selection bias and ensures the quality of sample selection for the DID estimator, and DID, which addresses possible endogeneity issues arising from PSM. The combination of the two models allows for more accurate estimation of the impact of the Broadband China pilot on occupational health. Specifically, we first divide the sample into two groups, the treatment group (pilot cities) and the control group (non-pilot cities). Secondly, as the control variables in this paper provide a good measure of the characteristics of each city, we use the control variables as matching variables in a Logit regression to predict the probability of piloting “Broadband China” policy in each sample city based on the regression results. Third, we then use PSM to match cities with similar predicted probabilities to obtain a control group of cities with similar characteristics to the treatment group. Fourth, on the basis of the above matching, we then use DID for estimation. Table 6 reports the estimation results of the PSM-DID. After using PSM to remove sample selection bias, we found that the coefficient of *DID* remain significantly negative and the matched estimates remained consistent with the baseline results. These results suggest that the baseline results are reliable, meaning that the digital economy can significantly improve occupational health.

**Table 6:** Results of PSM–DID model.

	**ln_rate**	**ln_death**	**ln_rate**	**ln_death**
	**(1)**	**(2)**	**(3)**	**(4)**
DID	−0.244^***^	−0.266^***^	−0.297^***^	−0.302^***^
	(0.050)	(0.047)	(0.048)	(0.049)
Control variables	×	×	√	√
Region FE	√	√	√	√
Year FE	√	√	√	√
Constant	0.235^***^	6.144^***^	5.298^***^	5.900^***^
	(0.045)	(0.042)	(0.703)	(0.707)
*R* ^2^	0.968	0.851	0.976	0.869
*N*	396	396	396	396

*** Indicates significance at the 1% level. Standard error in parentheses.

### Heterogeneity test

Hypothesis 2 assumes regional differences in the impact of the digital economy on occupational health. In areas with higher economic development, the effects of the digital economy on occupational health are more obvious. To test Hypothesis 2, we divide the whole sample into different groups. The first group is divided into a high-level group and a low-level group according to the level of the city's economic development. If the GDP of a city is less than the median GDP of all cities, then the city is classified into the low-level group. Otherwise, it is classified into the high-level group. The second group is divided into PRD cities and non-PRD cities according to whether they belong to the PRD region. According to the “Outline of the Reform and Development Plan for the Pearl River Delta region (2008–2020)” issued in 2009, we demarcated the regional scope. Nine cities, Guangzhou, Shenzhen, Zhuhai, Foshan, Jiangmen, Dongguan, Zhongshan, Huizhou, and Zhaoqing, are classified as PRD, and the remaining 12 cities, Chaozhou, Shantou, Jieyang, Shanwei, Meizhou, Zhanjiang, Maoming, Yangjiang, Yunfu, Qingyuan, Heyuan, and Shaoguan, are classified as non-PRD. After the groups were set, we incorporated the four groups of samples into the model and obtained Table 7.

**Table 7:** Results of heterogeneity test.

	**High-level group**		**Low-level group**		**PRD group**		**Non-PRD group**	
	**ln_rate**	**ln_death**	**ln_rate**	**ln_death**	**ln_rate**	**ln_death**	**ln_rate**	**ln_death**
	**(1)**	**(2)**	**(3)**	**(4)**	**(5)**	**(6)**	**(7)**	**(8)**
DID	−0.118^*^	−0.111^*^	−0.451^***^	−0.442^***^	−0.101^*^	−0.091	−0.461^***^	−0.464^***^
	(0.061)	(0.061)	(0.131)	(0.131)	(0.059)	(0.058)	(0.093)	(0.094)
Control variables	√	√	√	√	√	√	√	√
Region FE	√	√	√	√	√	√	√	√
Year FE	√	√	√	√	√	√	√	√
Constant	6.266^***^	6.245^***^	7.423^***^	8.032^***^	6.071^***^	6.407^***^	4.551^***^	5.055^***^
	(1.733)	(1.729)	(0.851)	(0.852)	(1.092)	(1.075)	(1.037)	(1.047)
*R* ^2^	0.971	0.877	0.975	0.854	0.989	0.939	0.971	0.845
*N*	199	199	200	200	171	171	228	228

^***^ and ^*^ respectively indicates significance at the 1% and 10% level. Standard error in parentheses.

As seen from Columns (1) to (4), only the estimation coefficient of “Broadband China” in the low-level group maintains a high level of significance, while that of “Broadband China” in the high-level group is not significant, and the absolute value of the coefficient of the former is much higher than that of the latter. Columns (5) to (6) also show that the estimated coefficient of “Broadband China” in the PRD sample is not significant, while in the non-PRD sample, the estimated coefficients of “Broadband China” are negative and significant at the 1% level in Columns (7) to (8). The above regression results indicate that the digital economy has a more obvious effect on occupational health in less developed areas compared with developed areas. Hence, we think Hypothesis 2 passes the test.

### Further mechanism testing

The above findings reveal the link between the development of the digital economy and the improvement of occupational health. In order to test the mechanism hypothesis, this paper selected two variables of industrial upgrading and technological innovation to test the mediation model combined with the conclusions of the existing literature. The specific supporting basis is as follows:

(1) Industrial upgrading. Industrial structure is an important factor affecting regional development (41). In China, some industrial manufacturing industries, such as metallurgy, non-ferrous metals, building materials, machinery, light industry, textile, tobacco and trade, have always been the areas with great occupational health risks, and have been listed as the key regulatory targets of the government. As digital economy and technology promote the development of modern service industry and advanced manufacturing industry in the regions (51), and the scale of the above industries with high occupational health risks is gradually reduced in the region relatively, which leads to less dangerous workplaces and risky work environments (44), and finally realizing the improvement of regional occupational health level. Therefore, industrial structure can be regarded as an important intermediate variable bridging the relationship between the development of digital economy and technology and the improvement of regional occupational health.

(2) Technological innovation. With the optimization of industrial structure, the adoption of new technologies by enterprises is also a potential factor to promote the reduction of occupational health risks and improve the health and wellbeing of employees, whether it's using digital technologies to optimize business management and increase productivity, Improving working conditions (28), Or specialized digital occupational health consulting, training and medical services (7), individual occupational health and welfare of the employees are able to benefit from. Therefore, the application of technology by enterprises is a non-negligible intermediate mechanism for digital economy and technology to improve occupational health.

Mechanism test results are as follows: The baseline regression result and robustness tests suggest that the digital economy is effective in improving occupational health, but the mechanisms at play are open to further discussion. We argued in the previous section that industrial upgrading (*Upg*) and technological innovation (*Inn*) are two very critical pathways through which the digital economy promotes occupational health. Next, we will test whether these two pathways exist through a mediating effects model. This paper draws on the step-by-step test to establish a mediating effect model (52). Firstly, this paper examines whether the digital economy has a significant impact on occupational health. Secondly, it tests whether the digital economy has a significant impact on industrial upgrading or technological innovation. Finally, digital economy and industrial upgrading or technological innovation are incorporated into the model at the same time to observe the changes of coefficients, as follows:


(5) $$\begin{array}{l} Y_{it}=\beta_{0}+\beta_{1}Broadband_{it}{+\sum \gamma_{j}X}_{it}+\mu_{i}+\sigma_{i}+\varepsilon_{it} \end{array}$$

(6) $$\begin{array}{l} M_{it}=\theta_{0}+\theta_{1}Broadband_{it}{+\sum \gamma_{j}X}_{it}+\mu_{i}+\sigma_{i}+\varepsilon_{it} \end{array}$$

(7) $$\begin{array}{l} Y_{it}=\eta_{0}+\eta_{1}Broadband_{it}{+M_{it}+\sum \gamma_{j}X}_{it}+\mu_{i}+\sigma_{i}+\varepsilon_{it} \end{array}$$

where, *M*_*it*_denotes mediating variables, including industrial upgrading and technological innovation. We calculate industrial upgrading using the following formula:


(8) $$\begin{array}{l} Upg=\left(\overset{3}{\underset{j=1}{\sum }}k_{j}\times \frac{p_{j}}{l_{j}}\right)j=1,2,3 \end{array}$$

where *j* = 1, 2, 3 denote the first, second and third industries respectively, *k*_*j*_ denotes the ratio of output value of industry *j* in total output value, *p*_*j*_ denotes the output value of industry *j*, *l*_*j*_ denotes the number of people employed in industry *j*, and $\frac{p_{j}}{l_{j}}$ characterizes the labor productivity of industry *j*. We choose to measure the innovation capacity of cities from the “Report on the Innovation Capacity of Chinese Cities and Industries” published by the Industrial Development Research Center of Fudan University of China.

Table 8 reports the results of the mediating effects test. As in the previous section, columns (1) and (2) based on equation (5) show that the coefficient on “Broadband China” is significantly negative. Columns (3) and (6) based on equation (6) show that the coefficient on “Broadband China” is positive and significant at the 10% level, implying that the digital economy can significantly contribute to industrial upgrading and technological innovation. We also include “Broadband China” and industrial upgrading in the model to obtain columns (4) and (5), and we find that the coefficient of industrial upgrading is significantly negative, and the absolute value of the coefficient of the variable “Broadband China” decreases significantly, indicating that industrial upgrading plays a significant role in the digital economy. This suggests that industrial upgrading plays a partially mediating role in the impact of the digital economy on occupational health. By including “Broadband China” and technological innovation in the model, we obtain columns (7) and (8), and we also find that the coefficient of the variable of technological innovation is significantly negative, and the absolute value of the coefficient of the variable of “Broadband China” also decreases significantly, implying that the development of the digital economy The coefficients of the variables for “Broadband China” also show a significant decrease in absolute value, implying that the development of the digital economy improves occupational health through technological innovation. In order to enhance the robustness, this paper also tested the mediating effect by Bootstrap method, and the results also supported the above conclusion.

**Table 8:** Mediation effect test.

	**ln_rate**	**ln_death**	**Upg**	**ln_rate**	**ln_death**	**Inn**	**ln_rate**	**ln_death**
	**(1)**	**(2)**	**(3)**	**(4)**	**(5)**	**(6)**	**(7)**	**(8)**
DID	−0.295^***^	−0.299^***^	0.939^**^	−0.285^***^	−0.288^***^	6.514^*^	−0.287^***^	−0.291^***^
	(0.048)	(0.048)	(0.455)	(0.048)	(0.048)	(3.512)	(0.048)	(0.048)
Upg				−0.011^*^	−0.012^**^			
				(0.006)	(0.006)			
Inn							−0.001^*^	−0.001^*^
							(0.001)	(0.001)
Control variables			√	√	√	√	√	√
Region FE			√	√	√	√	√	√
Year FE			√	√	√	√	√	√
Constant	5.298^***^	5.901^***^	−60.927^***^	4.657^***^	5.182^***^	49.341	5.361^***^	5.962^***^
	(0.701)	(0.705)	(6.694)	(0.776)	(0.780)	(51.630)	(0.700)	(0.704)
*R* ^2^	0.976	0.870	0.872	0.976	0.871	0.196	0.976	0.871
*N*	399	399	399	399	399	399	399	399

^***^, ^**^, and ^*^ respectively indicates significance at the 1% and 10% level. Standard error in parentheses.

## Discussion and conclusions

Our study provides exploratory knowledge contribution to verify and measure the positive health effects of the digital economy. This paper explores the relationship between the digital economy and occupational health and examines the regional heterogeneity of the relationship. The findings provide evidence of the health effects of the digital economy. Compared with the existing research in the field of occupational health, our paper forms several marginal theoretical contributions and policy implications as follows.

### Implication for studies of the health effects of digital economy

First, this paper explored the relationship between digital development and occupational health. Previous research of occupational health literature rarely involves the influence of the economic and industrial policy. Digital development is accompanied by advances in digital technologies that help improve people's health experience and have the potential to further enhance their health (2), which reflects a core marginal theoretical contribution of the paper. Meanwhile, we also verified a mechanism of digital economy in promoting occupational health, by introducing industrial upgrading and technological innovation as intermediate variables, our empirical findings support the link between the development of the digital economy and occupational health improvement, and also provide a reasonable explanation for the relationship. That is, the research branch line of the influence path of digital economy and technology—industrial upgrading and advanced technology application—occupational health improvement (28) mentioned in the previous literature review has been proven by the research work of the paper.

Second, another marginal contribution of this paper is to apply from the Chinese economy developed province, Guangdong province, a typical region of panel data to verify the existence of these relations. Based on exploring the health effects of China's digital economy, the regional distribution heterogeneity is found, that is, the marginal diminishing effect of the health effects of digital economy. First, this discovery conforms to the diffusion law and effect of a new technology application or emerging industry. Due to the early application of digital technology in the development of the new economy, the space for improvement of occupational health in the economically developed area or “PRD region” affected by the development of digital economy is shrinking, and the economically underdeveloped area or “non-PRD region” with slow development of digital economy may be in the “bonus period” of the health effect of digital economy. In addition, we reasonably speculate that the effect is related to the industrial structure and economic development factors in different regions. In the economically developed area or “PRD region,” the new economy represented by digital economy is developing rapidly and leading the country, at the same time, the local region also has more advanced employment mode and structure and has a higher-end talent employment market. But economically underdeveloped area or “non-PRD region” remains an industrial structure which is relatively backward, the traditional machining manufacturing which has higher occupational health risks in the economy are still very high share, as a result, the occupational health status of the economically underdeveloped area or “non-PRD region” is inherently worse than that of the economically developed area or “PRD region,” so the local occupational health improvement is better affected by the booming digital economy.

Third, empirical samples of spillover effects of economic and industrial policies on regional health under the background of strong government intervention are provided. China is the world's largest developing country and the world's second largest economy. With the increasingly widespread application of digital economy in various industries, China is no longer a simple follower but a “leader” in a specific field (53). This trend is closely related to the Chinese government's economic and industrial policies. In fact, consciously cultivating new technologies and industries has become “the hand of the intervention” for the authoritarian regime to develop the national economy. In the environment of strong heterogeneity, such as China, powerful and distinctive industry and science and technology policy intervention makes national digital (especially in the digital economy) get rapid and great development (18), it is a fact that cannot be ignored. And one of the knowledge marginal contributions of this paper is to present evidence of spillover effects of the above “government intervention” on improving occupational health in the region and provided a characteristic sample from China for related research.

### Policy recommendations for the government

In the modern society with increasing complexity and uncertainty, public health policy makers need to consider more influencing factors beyond the health management system to ensure the effectiveness and public welfare of health policy. This paper provides a thinking direction for policy practice of improving occupational health from the aspects of industrial and technological policy, regional endowment, and economic structure. In brief, this paper provides two policy implications:

First, vigorously develop new technologies and industries that are conducive to improve occupational health. The empirical results of this paper show that promoting the digitalization of regional economy and society has a significant spillover effect on improving regional occupational health. Therefore, the government should enlarge the coverage of broadband network and other digital infrastructure, improve the network of regional broadband hardware and software infrastructure construction, promote the digital technology and the application of digital economy in the field of occupational health, insure the public health policy information can more quickly and in a timely manner to reach high occupational health risk, and provide other digital health management services, to improve their health management experience, thus enhancing regional occupational health.

Second, develop digital occupational health intervention policies with regional differences. Considering the different impacts of digital economy development level on occupational health in different regions, local governments should formulate more detailed digital economy development goals and corresponding plans according to national or upper-level government policies and local economic development realities, and appropriately balance the digital economy development level in different regions. Digital economy should be linked with regional public health, especially occupational health policies, and differentiated occupational health policies should be formed on this basis, to more effectively stimulate the positive spillover effects of new industries and technologies such as digital economy and digital technology on regional occupational health level.

## Data availability statement

The original contributions presented in the study are included in the article/supplementary material, further inquiries can be directed to the corresponding author.

## Author contributions

Conceptualization, software, and formal analysis: FW. Methodology, writing—original draft preparation, and visualization: FW and ZW. Writing—review and editing and supervision: ZW. All authors have read and agreed to the published version of the manuscript.
